# Role of Baseline Computed-Tomography-Evaluated Body Composition in Predicting Outcome and Toxicity from First-Line Therapy in Advanced Gastric Cancer Patients

**DOI:** 10.3390/jcm10051079

**Published:** 2021-03-05

**Authors:** Silvia Catanese, Giacomo Aringhieri, Caterina Vivaldi, Francesca Salani, Saverio Vitali, Irene Pecora, Valentina Massa, Monica Lencioni, Enrico Vasile, Rachele Tintori, Francesco Balducci, Alfredo Falcone, Carla Cappelli, Lorenzo Fornaro

**Affiliations:** 1 Unit of Medical Oncology, Azienda Ospedaliero-Universitaria Pisana, Via Roma 67, 56126 Pisa, Italy; catanesesilvia@gmail.com (S.C.); f.salani1@gmail.com (F.S.); valentinamassa22@gmail.com (V.M.); monicalencioni65@gmail.com (M.L.); envasile@gmail.com (E.V.); alfredo.falcone@med.unipi.it (A.F.); lorenzo.fornaro@gmail.com (L.F.); 2 Department of Translational Research and New Surgical and Medical Technologies, University of Pisa, Via Savi 6, 56126 Pisa, Italy; giacomo.aringhieri@unipi.it (G.A.); racheletintori@hotmail.com (R.T.); francescocbalducci@gmail.com (F.B.); 3 Diagnostic and Interventional Radiology, Azienda Ospedaliero-Universitaria Pisana, Via Roma 67, 56126 Pisa, Italy; vitalisaverio@gmail.com (S.V.); carlacappelli1@gmail.com (C.C.); 4 Unit of Medical Oncology, Ospedale della Misericordia di Grosseto, Azienda Usl Sud Est, Via Senese 161, 58100 Grosseto, Italy; irene.pecora@gmail.com

**Keywords:** gastric cancer, metastatic, body composition, sarcopenia, visceral fat area, subcutaneous fat area, outcome, toxicity

## Abstract

Sarcopenia is recognised as a predictor of toxicity and survival in localised and locally advanced gastric cancer (GC). Its prognostication power in advanced unresectable or metastatic GC (aGC) is debated. The survival impact of visceral and subcutaneous fat distribution (visceral fat area (VFA)/subcutaneous fat area (SFA)) is ambiguous. Our aim was to determine the influence of body composition parameters (BCp) on toxicity and survival in aGC patients undergoing palliative treatment. BCp were retrospectively assessed by baseline computed tomography for 78 aGC patients who received first-line chemotherapy from March 2010 to January 2017. Correlations between BCp and toxicity and survival were calculated by χ^2^-test and by log-rank-test and Cox-model, respectively. Sarcopenia fails to show association with progression-free survival (PFS) (*p* = 0.44) and overall survival (OS) (*p* = 0.88). However, sarcopenia influences the development of high-grade neutropenia (*p* = 0.048) and mucositis (*p* = 0.054). VFA/SFA (high vs. all the rest) results as a strong predictor of objective response (*p* = 0.02) and outcome (PFS, *p* = 0.001; OS, *p* = 0.02). At multivariate analysis for PFS, prognostic factors are VFA/SFA (*p* = 0.03) and a neutrophil–lymphocyte ratio >3. The same factors remain significant for OS (each *p* = 0.03) along with Eastern Cooperative Oncology Group (ECOG) performance status (*p* = 0.008) and number of metastatic sites ≥2 (*p* < 0.001). In our cohort of aGC, VFA/SFA exhibit a robust impact on survival, with a higher sensitivity than sarcopenia.

## 1. Introduction

Gastric cancer (GC) is the fifth most common malignancy and the third leading cause of cancer death worldwide [1]. Sixty percent of cases are inoperable or advanced at diagnosis (aGC), requiring palliative chemotherapy treatment [2]. Body weight changes are common in patients with aGC, even though they do not strictly correlate with body composition changes. The body of literature investigating the prognostic role of body composition has progressively increased in recent years [3]. The actual gold standard to evaluate skeletal muscle mass and adipose tissue distribution variations is the analysis of a computed tomography (CT) scan at the level of the third lumbar vertebra (L3) [4,5]. The depletion of skeletal muscle mass (sarcopenia) has been recognised as a poor outcome predictor in many solid tumours and in localised and locally aGC [6,7,8,9,10,11]. Moreover, after gastrectomy, baseline preoperative sarcopenia constitutes an independent risk factor for postoperative surgical complications and infections [9,10,11]. Despite this evidence, in the case of advanced disease, the impact of sarcopenia remains controversial [12,13,14,15,16]. The association with treatment-related toxicity has been mainly investigated in the perioperative setting: in two retrospective series, a trend toward a moderately positive correlation with treatment reduction, postponement or discontinuation was observed [17,18]. Though data are not conspicuous, sarcopenia was not confirmed as a predictor of toxicity in the metastatic setting [12,15]. The alteration of adipose tissue distribution among the visceral and the subcutaneous compartment is a known metabolic disruption occurring during the progression of neoplastic disease [19]. The ratio between visceral fat area (VFA) and subcutaneous fat area (SFA) has shown a negative prognostic impact in many retrospective series in gastrointestinal cancers, despite the fact that the majority were in the preoperative or perioperative setting [20,21,22,23,24]. 

Considering the poor prognosis of aGC, a further exploration of these parameters appeared necessary to clarify their role as outcome and toxicity predictors and improve our quality care. The aim of our work was to define which body composition parameter better correlates with outcome and chemotherapy-derived toxicity in a homogeneous cohort of Caucasian aGC patients treated with first-line palliative chemotherapy. 

## 2. Materials and Methods

### 2.1. Study Population

**Figure A1:**
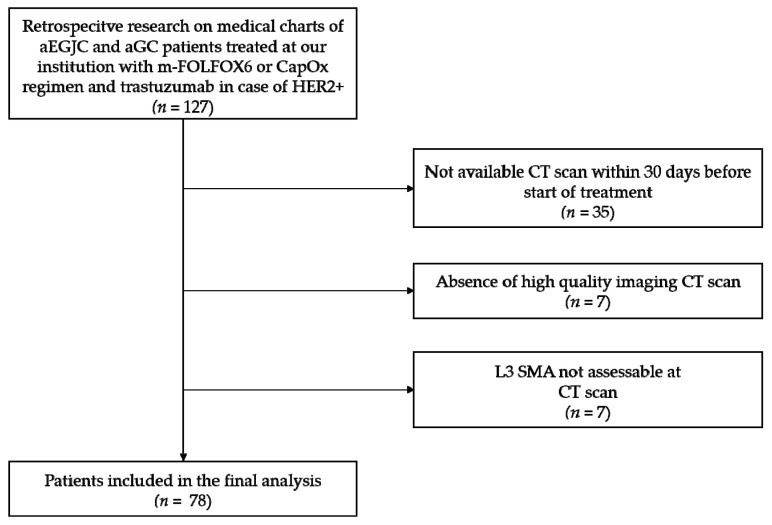
Patient’s selection flow-chart. Abbreviations: aEGJC, advanced oesophagogastric junction cancer; aGC, advanced gastric cancer; HER2, epidermal growth factor receptor 2; CT, computed tomography; L3 SMA, third lumbar vertebra skeletal muscle area.

We retrospectively evaluated the medical records of consecutive patients diagnosed with advanced esophagogastric junction carcinoma or aGC, who received at least one cycle of first-line doublet chemotherapy at our Azienda Ospedaliero-Universitaria Pisana from March 2010 to January 2017. All selected cases had a histologically proven diagnosis of adenocarcinoma. Patients whose CT scans lacked images of the third lumbar vertebra within 30 days prior to treatment initiation or patients who had palliative systemic therapy before CT evaluation were excluded (study inclusion flow-chart, Figure A1). Collected clinicopathological data included: age, sex, height, weight, Eastern Cooperative Oncology Group (ECOG) performance status (PS), primary tumour and metastatic sites, previous treatment history, human epidermal growth factor receptor 2 (HER2) status, baseline laboratory values (complete blood count with differential count and serum chemistry), chemotherapy regimen and administration, radiological response, survival status and last follow-up. Neutrophils–lymphocytes ratio (NLR) and platelets–lymphocytes ratio (PLR) were also collected at baseline (before cycle one administration) and dichotomised according to literature data cut-offs for metastatic gastric cancer patients: NLR > vs. ≤ 3 and PLR > vs. ≤ 200 [25,26].

All patients signed institutionally approved written informed consent before treatment administration.

### 2.2. Treatment

Only patients who received standard first-line palliative systemic therapy were included. Treatments consisted of the combination of fluoropyrimidine and platinum compound according to modified FOLFOX-6 regimen (mFOLFOX6) (5-FU 400 mg/m^2^ bolus on day 1 and 2400 mg/m^2^ continuous infusion from day 1 to 3 plus oxaliplatin 85 mg/m^2^ on day 1, in a two-weekly cycle) and CapOX regimen (capecitabine 1000 mg/m^2^, taken orally two times a day on days 1 to 14 plus oxaliplatin 130 mg/m^2^ on day 1, in a three-weekly cycle). 5-FU was preferred over capecitabine in the case of dysphagia or contraindication to oral fluoropyrimidine (such as concomitant treatment with oral anticoagulants), while capecitabine was preferred in case of a patient’s request for an oral treatment. In case of HER2-positive disease, trastuzumab was added to the chemotherapy backbone. Treatment was discontinued in case of disease progression, unacceptable toxicity, or on patient’s request. Toxicity was assessed using the Common Terminology Criteria for Adverse Events (version 4.03) by recording the highest grade of each adverse event throughout all administered cycles [27]. In the case of the development of neurotoxicity of high grade (G3–G4), according to CTCAE oxaliplatin administration was discontinued and maintenance with fluoropyrimidine monotherapy was provided.

### 2.3. Efficacy and Outcome

Response evaluation was performed according to Response Evaluation Criteria in Solid Tumours version 1.1 (RECIST 1.1), using radiological follow-up assessments obtained every 8 to 12 weeks during treatment [28]. Progression-free survival (PFS) and overall survival (OS) were defined as the time from first-line chemotherapy initiation to the date of radiological/clinical progression or death from any cause and to the date of death or last follow-up, respectively. 

### 2.4. Body Composition Parameters Assessment

Slice thickness of included CT exams ranged from 2.5 to 3 mm. To evaluate the quantitative assessment of skeletal muscle mass, visceral and subcutaneous fat tissue for each patient, portal phase CT images, performed prior to the initiation of therapy, were analysed by a trained radiologist who was blinded to clinical data on GE advantage workstation (software version 4.7, G.E. Healthcare, Milwaukee, WI, USA).

Measurements of the total muscle areas were made on transverse images at the third lumbar vertebra (L3) level, with the transverse processes fully visible. First, visceral and subcutaneous fat was segmented, and VFA and SFA were separately calculated. Then, after visceral and subcutaneous fat removal, the skeletal muscle area was measured using automatic tissue-specific Hounsfield unit (HU) thresholds, according to literature values (−50 to 140 HU).

The VFA/SFA ratio was calculated for each patient. Since no specific thresholds are known to define normal VFA/SFA values, we divided the study population into quartiles, as previously performed by other groups in oesophageal cancers [23]. Quartiles (Q) were defined as follows: Q1 the lowest, Q4 the highest. In addition, the VFA/SFA variable was treated as dichotomous with Q4 being defined as the “high ratio” and Q1–3 as “all the rest ratio”.

Sarcopenia was categorized according to the Martin cut-off values for Skeletal Muscle Index (SMI, defined as Skeletal Muscle Area (SMA) measured at L3 vertebra normalized for height squared), considering sex and body mass index (BMI), which demonstrated a strong correlation with poor outcome in a large cohort of patients affected by gastrointestinal and respiratory tract tumours. In detail, sarcopenia was defined in male patients as SMI < 43 cm^2^/m^2^ if BMI < 25 kg/m^2^ and SMI < 53 cm^2^/m^2^ if BMI ≥ 25 kg/m^2^, and in female patients as SMI < 41 kg/m^2^ irrespective of BMI [6]. 

### 2.5. Statistical Analysis

Descriptive statistics were provided as the proportion or medians with standard deviations and ranges for continuous variables. The association of sarcopenia, and different VFA/SFA ratio cut-offs with clinicopathological parameters, efficacy and toxicity were performed using the Pearson’s χ^2^ test or Fisher’s exact test for categorical variables. Continuous variables were analysed with the Mann–Whitney U test. PFS and OS were estimated applying the Kaplan–Meyer method and compared by the mean of the log-rank test. A multivariable Cox proportional hazard model was built to identify prognostic predictors of outcome: only factors with a two-sided *p*-value < 0.05 by the log-rank test were included. The analysis was performed using the statistical software Medcalc version 14.8.1 (Medcalc, Ostend, Belgium).

## 3. Results

### 3.1. Patient Characteristics and Body Composition Parameters Distribution

Characteristics of the 78 patients included and the correlations with body composition variables are depicted in Table 1. Median age was 67 years (range 35–80). Most of the patients (72%) were male and presented more than two metastatic sites (62%) of which lymph nodes and peritoneum had the highest frequency. Sixteen patients (24%) were HER2 positive. At baseline, 47% of patients were of normal weight, only 6% were obese, according to BMI categories.

#### 3.1.1. Skeletal Muscle

The mean L3 SMI was 40.65 cm^2^/m^2^ (range: 25.48–61.94) for females and 48.51 cm^2^/m^2^ (range: 32.73–68.70) for males. As per Martin’s cut-off values, 34% of patients were judged to be sarcopenic. No significant associations were observed between sarcopenia and clinico–pathological characteristics, except for a higher prevalence of bone metastasis in patients with a loss of muscle mass (*p* = 0.05). 

#### 3.1.2. Adipose Tissue

Regarding fat distribution parameters, assessable in 57 patients, the median VFA was 89.10 cm^2^ (range: 3.56–407.77), the median SFA was 108.99 cm^2^ (range: 0.88–355.97) and the median VFA/SFA ratio was 1.09 (range: 0.17–4.05). According to quartiles of the VFA/SFA ratio, patients were classified as follows: first quartile cases (Q1: ≤0.58); second quartile cases (Q2: 0.59–1.09): third quartile cases (Q3: 1.10–1.53); and fourth quartile cases (Q4: ≥1.54). Due to the findings of efficacy and survival for each Q group (see Section 3.2.1, Figure A3a,b and Table A1), we defined a dichotomous VFA/SFA ratio: group Q4 as the “high VFA/SFA” and groups Q1–3 as the “all the rest VFA/SFA”. Only fourteen patients presented a high VFA/SFA ratio. All clinicopathological characteristics were stratified and analysed in the aforementioned groups. Significant differences in terms of the presence of ≥2 metastatic sites and especially lymph nodes metastasis among the high VFA/SFA ratio group was noticed (*p* = 0.02).

### 3.2. Body Composition Parameters and Outcome 

#### 3.2.1. Efficacy

At a median follow-up of 52.2 months (range: 31.25–87.66), 74 patients (95%) had progressed, and 70 (90%) had died: median PFS (mPFS) was 5.9 months (95% CI: 4.8–7.2) and median OS (mOS) was 10.8 months (95% CI: 9.5–12.9).

**Figure A2:**
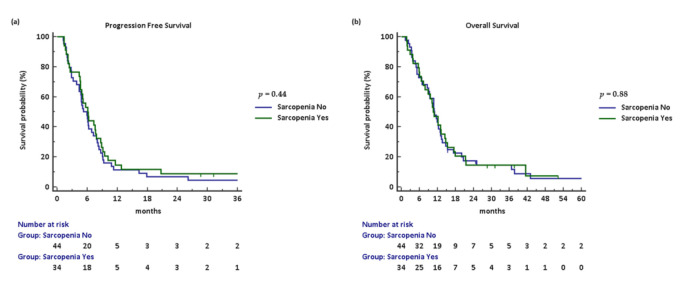
Kaplan–Meyer curves according to presence or absence of sarcopenia: (**a**) progression-free survival; and (**b**) overall survival.

Neither PFS (hazard ratio (HR): 0.83, 95% CI: 0.53–1.32; *p* = 0.44) nor OS (HR: 0.97, 95% CI: 0.60–1.55; *p* = 0.88) differed between sarcopenic and non-sarcopenic patients (Figure A2).

**Figure 1:**
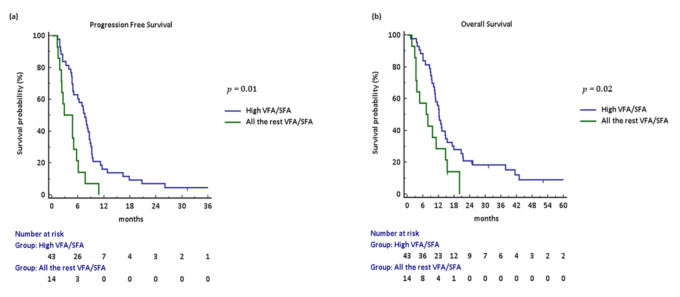
Kaplan–Meyer curves according to VFA/SFA ratio: (**a**,**b**) progression-free and overall survival stratified by high vs. all the rest, respectively. Abbreviations: VFA/SFA, visceral fat area/subcutaneous fat area.

**Figure A3:**
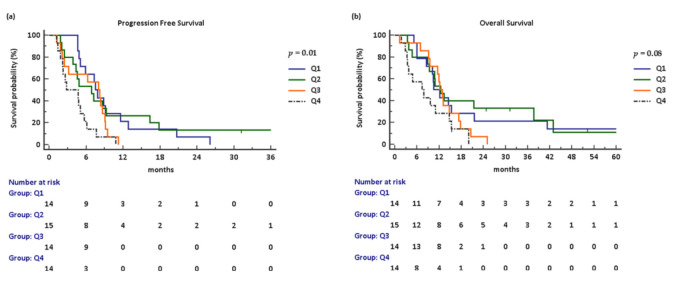
Kaplan–Meyer curves according to the VFA/SFA ratio: (**a**,**b**) progression-free and overall survival stratified by quartiles, respectively.

As shown in Figure A3a,b, Kaplan–Meyer curves were calculated for each Q group of the VFA/SFA ratio. A statistically significant worsening of PFS (*p* = 0.01) and a trend toward worse OS (*p* = 0.08) were observed for Q4 compared to any other group in a univariate Cox proportional hazards model (Table A1). Therefore, we proceed to analyse the VFA/SFA ratio as dichotomous groups: Q1–3 as “all the rest VFA/SFA ratio” and Q4 as the “high VFA/SFA ratio”. Looking at Q1–3 vs. Q4 survival comparison, patients in the high VFA/SFA group experienced a statistically significant worse PFS (HR: 2.49, 95% CI: 1.11–5.61; *p* = 0.001) and OS (HR: 2.02, 95% CI: 0.93–4.41; *p* = 0.02) compared to those in the all the rest of the VFA/SFA group (Figure 1a,b). 

#### 3.2.2. Activity

Partial response (PR) was achieved in 28 patients (no complete responses were observed; response rate, RR: 35.9%), and disease control was achieved in 55 patients (disease control rate, DCR: 70.5%). 

The presence of sarcopenia did not affect activity (PR vs. stable disease (SD) vs. progressive disease (PD): *p* = 0.55) or DCR (*p* = 0.44).

**Figure 2:**
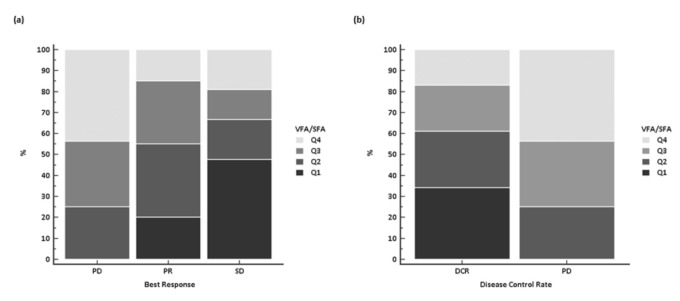
Response according to the VFA/SFA ratio, stratified by quartiles: (**a**) best response; and (**b**) disease control rate. Abbreviations: VFA/SFA, visceral fat area/subcutaneous fat area; Q, quartile; PD, progressive disease; PR, partial response; SD, stable disease; DCR, disease control rate.

Conversely, a significant difference in treatment activity was noticed among the 57 patients belonging to different VFA/SFA quartiles ratio, with no PD observed in Q1 group in contrast to PD probability of 50% in the Q4 group (*p* = 0.02). The statistically significant difference was retained also when responses were compared as presence vs. absence of DCR (*p* = 0.03) (Figure 2).

### 3.3. Cox Proportional Hazards Model for Survival of Body Composition and Clinical Parameters

#### 3.3.1. Univariate Analysis

As previously discussed, at univariate analysis, sarcopenia showed no prognostic impact, while the VFA/SFA ratio as a dichotomous variable was strongly prognostic for both PFS and OS. ECOG PS was highly prognostic in the log-rank test both for PFS (*p* = 0.02) and OS (*p* = 0.03). The presence of bone lesions represented the strongest clinical predictor of OS (*p* < 0.001). Systemic inflammatory parameters such as NLR and PLR confirmed their prognostic impact on OS (*p* = 0.007 and *p* = 0.04, respectively), with NLR also being associated with PFS (*p* = 0.008) (Table 2). 

#### 3.3.2. Multivariate Analysis

At multivariate analysis, the VFA/SFA ratio retained its prognostic role for PFS (*p* = 0.03) and OS (*p* = 0.02), as did NLR for both survival parameters (each *p* = 0.03). ECOG PS (0 vs. 1–2) and the presence of bone metastases maintained their independent value for OS (*p* = 0.008 and *p* < 0.001, respectively) (Table 3).

### 3.4. Body Composition Parameters as Toxicity Predictors

#### 3.4.1. Treatment Exposure

Thirty percent of patients received at least six cycles of chemotherapy, 8 being the median number (range 1–12). In 36 patients (46.2%), the doses of fluoropyrimidine and/or oxaliplatin were reduced or treatment administration was postponed due to toxicity. Treatment was discontinued due to adverse events in six patients (7.7%).

Chemotherapy administration was not significantly influenced by the presence of sarcopenia (*p* = 0.29) or visceral/subcutaneous adipose tissue distribution alterations (*p* = 0.95). 

#### 3.4.2. Adverse Events

A grade 3–4 hematologic adverse event was reported in 27 patients (35%). Grade 3–4 non-hematologic adverse events affected only nine patients (12%). 

Sarcopenia seemed to be significantly associated with grade 3–4 neutropenia (*p* = 0.048): among patients who suffered from grade 3–4 neutropenia, 61.9% were sarcopenic compared to 18.2% who did not present muscle mass reduction. The development of mucositis of any grade was also significantly associated with sarcopenia (55.9 vs. 44.1%, *p* = 0.054). VFA/SFA ratios did not show any correlation with toxicity.

## 4. Discussion

In our study, sarcopenia was observed in nearly half of the enrolled population and did not affect the response and survival of aGC patients treated with first-line doublet chemotherapy. Indeed, sarcopenia had an impact on the development of grade 3–4 hematologic toxicity and any grade mucositis. Of note, a higher proportion of visceral fat over subcutaneous fat was convincingly associated with an unfavourable prognosis, without showing the influence on treatment tolerance. 

The impact of sarcopenia on toxicity has been to date scarcely investigated in first-line aGC: almost no association was found in the literature, except for a correlation between baseline sarcopenic obesity and grade 2–4 neurotoxicity [12,15]. The body of evidence in the perioperative setting suggests a significant association between sarcopenia and dose-limiting toxicity and early treatment termination [17,18]; in contrast, we did not find a negative impact of sarcopenia on treatment compliance and exposure. The correlation we identified with high-grade neutropenia in sarcopenic patients could possibly be explained by the known association with lean and muscle mass with pharmacokinetics parameters such as drug distribution, metabolism and the clearance of chemotherapeutic agents, especially for hydrophilic ones such as fluoropyrimidines [8,29]. 

Although several studies reported an association between the loss of skeletal muscle mass and OS [6,7,9,12,14], we did not observe such a correlation. This was possibly due to the reduced power of the study and the relatively small number of obese patients (6%, Table 1), considering that the survival prediction of Martin’s cutoffs was greater in overweight and obese patients [6]. In line with our data, two recent studies in the same setting and in a Caucasian population achieved comparable negative results in terms of OS. Dijksterhuis et al. observed no association between skeletal muscle and survival in advanced esophagogastric cancer patients receiving palliative chemotherapy. In an extensive retrospective analysis of baseline CT scans from the phase 3 EXPAND trial, sarcopenia was identified as a predictor only for PFS [15,16]. 

Rather than skeletal muscle quantity, the quality of skeletal muscle mass appears to be relevant for survival. Of uttermost importance, this can be studied on CT scan: the skeletal muscle density (SMD) and the mean muscle attenuation (MA) are both parameters of skeletal muscle infarction by adipose tissue, which compromises muscle properties. This issue has been primarily investigated by a Japanese retrospective study on aGC that showed lower SMD as independent predictor of survival in association with more than two metastatic sites [13]. Interestingly, Hacker et al., in the analysis of 761 radiological and clinical data from advanced esophagogastric cancer patients treated with first-line chemotherapy achieved similar conclusions: MA constituted the only powerful body composition parameter with a prognostic value for OS, although large differences in MA were translated into only moderate differences in an expected cohort [16].

Considering that reduced SMD and/or MA, whose prognostic impact has been extensively discussed, are consequences of higher cytoplasmatic depots of intramyocellular lipid droplets as well as intermuscular adipocytes [30], we might infer that the impact on outcome observed for VFA/SFA in our work should be inscribed in the same context. 

The role of baseline visceral and subcutaneous fat amount and distribution has been explored in the operable setting in localised colorectal cancer patients undergoing surgery, and in locally advanced rectal cancer planned to receive neoadjuvant chemoradiotherapy: a correlation of higher VFA/SFA ratio with poorer disease-free survival (DFS) was demonstrated [20,21]. The same results concerning these fat parameters were obtained in patients affected by squamous oesophageal cancer after esophagectomy [23]. In a single centre study cohort from the phase 3 CLASSIC (Capecitabine and Oxaliplatin Adjuvant Study in Stomach Cancer) trial, the marked loss of VFA and SFA, analysed as indexes normalized by height squared (VFI and SFI, respectively), appeared as a poor prognostic factor for DFS both in the group receiving adjuvant chemotherapy, and in the surgery-only group; the negative correlation with OS was demonstrated only in the interventional arm [24]. This last evidence was in line with a retrospective evaluation of preoperative body composition parameters in 507 upper gastrointestinal cancer patients: in this study, low visceral fat cases experienced a higher overall mortality rate [22]. However, the results of these two works should not be considered to be conflicting with the other literature evidence and our findings. In fact, adipose tissue parameters were investigated as single entities, instead of a proportion of visceral and subcutaneous fat components. 

Chronic insulin resistance is a known metabolic disruption occurring in malignant tumours in early stages prior to the development of weight loss and cachexia, actively contributing to its pathogenesis [19]. Body composition parameters and especially visceral adipose tissue have been shown to significantly correlate with insulin signalling [31]. The adipose tissue is an endocrine organ, secreting adipocytokines like adiponectin and leptin and cytokines (IL-1, IL-6, TNF-α), which regulates appetite, inflammation, insulin sensitivity and fat metabolism itself. Excess of adipose tissue, particularly the visceral component, metabolically more active than the subcutaneous one, is strongly associated with inflammatory cytokines production, the upregulation of nuclear factor-kB leading to increased nitric oxide and reactive oxygen species, which further propagate inflammation. Thus, visceral adipose tissue activity along with insulin resistance, and systemic inflammation would promote and perpetuate a pro-tumorigenic environment [19]. In this context, our findings appear convincing and we should highlight the point that VFA/SFA is independently associated with response, PFS and OS. Moreover, at multivariate analysis, the only other factor that retained significance for PFS was the inflammation parameter NLR. Lastly, along with known prognostic factors for OS like ECOG PS and a higher number of metastatic sites, VFA/SFA and NLR maintained their prognostic role in the multivariate model. Due to the mentioned evidence, we suggest that the VFA/SFA ratio is the best factor that depicts the metabolic changes occurring during cancer initiation and progression.

We are aware of the limitations of our study represented by its retrospective design, the single centre recruitment, and the small sample size of enrolled patients. Nevertheless, it represents a homogeneous aGC population of Caucasian patients treated with the actual standard of care in first-line doublet chemotherapy.

## 5. Conclusions

Our study confirms the absence of a prognostic role for sarcopenia in the advanced setting and shows the VFA/SFA ratio, readily assessable during routine radiological exams, as a potential game changer in the natural history of aGC. Further investigation on this putative prognostic biomarker is warranted, along with the exploration of new nutritional, hormonal and/or anti-inflammatory interventions (for example adiponectin replacement) to better clarify its possible role as a therapeutic target. 

## Figures and Tables

**Table 1:** Patient characteristics according to loss of muscle mass and abdominal fat distribution.

Characteristics	All Patients	Sarcopenia		*p*-Value	VFA/SFA		*p*-Value
	(*n* = 78) *N* (%)	Yes (*n* = 34) *N* (%)	No (*n* = 44) *N* (%)		All the Rest (*n* = 43) *N* (%)	High (*n* = 14) *N* (%)	
Age, years							
Median, (range)	67, (35–80)	70, (35–80)	66, (37–79)	0.29	66, (35–80)	65, (55–78)	0.87
Sex							
Female/male	22 (18)/56 (72)	12 (35)/22 (65)	10 (23)/34 (77)	0.33	13 (30)/30 (70)	2 (14)/12 (86)	0.40
ECOG PS							
0 vs. 1–2	34 (44)/44 (56)	12 (35)/22 (65)	22 (50)/22 (50)	0.28	23 (54)/20 (46)	4 (29)/10 (71)	0.19
Primary tumour site							
EGJ/PGC/DGC	32 (41)/26 (33)/20 (27)	13 (38)/10 (30)/11 (32)	19 (43)/16 (36)/9 (20)	0.48	12 (28)/21 (49)/10 (23)	8 (58)/3 (21)/3 (21)	0.10
Primary tumour surgery							
Yes/no	22 (28)/56 (72)	31 (91)/3 (9)	41 (93)/3 (7)	0.95	15 (35)/28 (65)	3 (21)/11 (79)	0.62
N° metastatic sites							
1 vs. ≥2	30 (38)/48 (62)	12 (35)/22 (65)	18 (41)/26 (59)	0.78	20 (46)/23 (54)	1 (7)/13 (93)	0.02
Metastatic sites							
Liver	30 (38)	11 (32)	19 (43)	0.46	20 (47)	5 (36)	0.69
Lung	6 (7)	4 (12)	2 (5)	0.45	2 (5)	3 (22)	0.16
Lymph nodes	52 (66)	24 (71)	28 (64)	0.68	24 (56)	13 (93)	0.02
Peritoneum	39 (50)	17 (50)	22 (50)	0.81	22 (51)	6 (43)	0.81
Bone	7 (9)	6 (18)	1 (2)	0.05	2 (5)	3 (21)	0.16
HER2 *							
Yes/no	16 (24)/51 (76)	7 (25)/21 (75)	9 (23)/30 (77)	0.91	10 (25)/30 (75)	2 (25)/6 (75)	0.65
NLR>3							
Yes/no/na	39 (50)/38 (49,9)/1(0,1)	18 (53)/15 (44)/1 (3)	21 (48)/23 (52)/–	0.71	21 (49)/22 (51)	8 (62)/5 (38)	0.62
PLR >200							
Yes/no/na	36 (46)/40 (51,8)/2 (0,2)	13 (38)/20 (59)/1 (3)	23 (52)/20 (45)/1 (3)	0.32	24 (57)/18 (43)	4 (31)/9 (69)	0.18
BMI							
≤20/20–24.9/25–30/≥30	17 (23)/37 (47)/19 (24)/5 (6)	–	–	–	–	–	–
SMI, median (range; SD)							
Female	40.65 (25.48–61.94; 8.55)	–	–	–	–	–	–
Male	48.51 (32.73–68.70; 8.50)	–	–	–	–	–	–
VFA ^§^, median (Range; SD)	89.10 (3.56–407.77; 88.57)	–	–	–	–	–	–
SFA ^§^, median (Range; SD)	108.99 (0.88–355.97; 80.55)	–	–	–	–	–	–

VFA/SFA, visceral fat area/subcutaneous fat area; ECOG PS, Eastern Cooperative Oncology Group (ECOG) performance status; EGJ, esophago–gastric junction cancer; PGC, proximal gastric cancer; DGC, distal gastric cancer; HER2, epidermal growth factor receptor 2; NLR, neutrophil–lymphocyte ratio; PLR, platelet–lymphocyte ratio; BMI, body mass index (kg/m^2^); SMI, skeletal muscle index (cm^2^/m^2^); VFA, visceral fat area (cm^2^); SFA, subcutaneous fat area (cm^2^) * Data available for 67 patients (pts); ^§^ Data available for 57 pts.

**Table 2:** Univariate analyses for progression-free survival and overall survival.

Variables	Progression-Free Survival		Overall Survival	
	HR (95% CI)	*p*-Value	HR (95% CI)	*p*-Value
Age				
≥ vs. < 67 years	0.85 (0.54–1.35)	0.47	0.73 (0.46–1.18)	0.18
ECOG PS				
0 vs. 1–2	0.58 (0.37–0.92)	0.02	0.59 (0.37–0.95)	0.03
Primary tumour surgery				
Yes vs. no	0.70 (0.43–1.13)	0.16	0.69 (0.42– 1.13)	0.16
N° metastatic sites				
1 vs. ≥2	0.64 (0.41–1.01)	0.06	0.63 (0.39– 1.01)	0.06
Metastatic sites				
Liver	0.91 (0.57–1.44)	0.68	0.67 (0.42–1.07)	0.09
Lung	1.56 (0.57–4.30)	0.29	1.27 (0.46–3.47)	0.61
Lymph nodes	1.21 (0.75–1.75)	0.43	1.31 (0.81–2.12)	0.29
Peritoneum	1.07 (0.67–1.68)	0.77	1.40 (0.87–2.24)	0.16
Bone	2.10 (0.72– 6.15)	0.05	4.35 (0.97–19.57)	<0.001
NLR > 3				
Yes vs. no	1.81 (1.13–2.90)	0.008	1.88 (1.16–3.05)	0.007
PLR > 200				
Yes vs. no	1.44 (0.90–2.30)	0.11	1.63 (1.00–2.65)	0.04
SMI				
Yes vs. no	0.83 (0.53–1.32)	0.44	0.96 (0.60–1.55)	0.88
VFA/SFA				
All the rest vs. high	0.40 (0.18–0.90)	0.002	0.49 (0.23–1.10)	0.02

Abbreviations: HR, hazard ratio; ECOG PS, ECOG performance status; NLR, neutrophil–lymphocyte ratio; PLR, platelet–lymphocyte ratio; SMI, skeletal muscle index; VFA/SFA, visceral fat area/subcutaneous fat area.

**Table 3:** Multivariate analysis for progression-free and overall survival.

Variables	Progression-Free Survival		Overall Survival	
	HR (95% CI)	*p*-Value	HR (95% CI)	*p*-Value
ECOG PS				
0 vs. 1–2	1.59 (0.90–2.81)	0.11	2.36 (1.25–4.44)	0.008
N° metastatic sites				
1 vs. ≥2	0.99 (0.52–1.87)	0.97	1.35 (0.67–2.63)	0.41
Metastatic sites				
Bone	2.32 (0.86–6.26)	0.09	9.63 (3.16–29.37)	<0.001
NLR>3				
Yes vs. no	2.00 (1.08–3.69)	0.03	2.19 (1.08–4.45)	0.03
PLR >200				
Yes vs. no	–	–	1.59 (0.80–3.19)	0.18
VFA/SFA				
All the rest vs. high	2.23 (1.08–4.59)	0.03	2.42 (1.44–5.13)	0.02

Abbreviations: HR, hazard ratio; ECOG PS, ECOG Performance Status; NLR, neutrophil to lymphocyte ratio; PLR, platelet to lymphocyte ratio; VFA/SFA, visceral fat area/subcutaneous fat area.

## Data Availability

All data are already presented in the manuscript.

## Appendix A

**Table A1:** Univariate analysis for progression-free and overall survival according to VFA/SFA ratio stratified by quartiles.

VFA/SFA Quartile	N°	Progression-Free Survival		Overall Survival	
		HR (95% CI)	*p*-Value	HR (95% CI)	*p*-Value
Q4 (≥1.54)	14	1 (Reference Value)	0.01	1 (Reference Value)	0.08
Q3 (1.10–1.53)	14	1.89 (0.74–4.84)	–	1.56 (0.63–3.88)	–
Q2 (0.59–1.09)	15	2.85 (1.16–6.97)	–	2.42 (1.02–5.78)	–
Q1 (≤0.58)	14	2.76 (1.14–6.71)	*–*	2.16 (0.89–5.21)	–

Abbreviations: VFA/SFA, visceral fat area/subcutaneous fat area; N°, number of cases; HR, hazard ratio; Q, quartile.
